# Behavioral and Electrocortical Response to a Sensorimotor Conflict in Individuals with Fibromyalgia

**DOI:** 10.3390/brainsci13060931

**Published:** 2023-06-08

**Authors:** Tania Augière, Martin Simoneau, Clémentine Brun, Anne Marie Pinard, Jean Blouin, Laurence Mouchnino, Catherine Mercier

**Affiliations:** 1 Center for Interdisciplinary Research in Rehabilitation and Social Integration (Cirris), Quebec City, QC G1M 2S8, Canada; tania.augiere.1@ulaval.ca (T.A.); martin.simoneau@kin.ulaval.ca (M.S.); anne-marie.pinard@fmed.ulaval.ca (A.M.P.); 2 Department of Rehabilitation, Faculty of Medicine, Laval University, Quebec City, QC G1V 0A6, Canada; 3 Department of Kinesiology, Faculty of Medicine, Laval University, Quebec City, QC G1V 0A6, Canada; 4 Department of Anesthesiology and Intensive Care, Faculty of Medicine, Laval University, Quebec City, QC G1V 0A6, Canada; 5 Laboratoire de Neurosciences Cognitives, Aix-Marseille University, National Center for Scientific Research (CNRS), 13331 Marseille, France; jean.blouin@univ-amu.fr (J.B.); laurence.mouchnino@univ-amu.fr (L.M.); 6 Institut Universitaire de France, 75005 Paris, France

**Keywords:** pain disorder, motor adaptation, sensorimotor integration, electroencephalography

## Abstract

People with fibromyalgia have been shown to experience more somatosensory disturbances than pain-free controls during sensorimotor conflicts (i.e., incongruence between visual and somatosensory feedback). Sensorimotor conflicts are known to disturb the integration of sensory information. This study aimed to assess the cerebral response and motor performance during a sensorimotor conflict in people with fibromyalgia. Twenty participants with fibromyalgia and twenty-three pain-free controls performed a drawing task including visual feedback that was either congruent with actual movement (and thus with somatosensory information) or incongruent with actual movement (i.e., conflict). Motor performance was measured according to tracing error, and electrocortical activity was recorded using electroencephalography. Motor performance was degraded during conflict for all participants but did not differ between groups. Time–frequency analysis showed that the conflict was associated with an increase in theta power (4–8 Hz) at conflict onset over the left posterior parietal cortex in participants with fibromyalgia but not in controls. This increase in theta suggests a stronger detection of conflict in participants with fibromyalgia, which was not accompanied by differences in motor performance in comparison to controls. This points to dissociation in individuals with fibromyalgia between an altered perception of action and a seemingly unaltered control of action.

## 1. Introduction

Our brain continuously integrates afferent information provided by our senses and efferent information, such as motor commands. This sensorimotor integration is essential for optimal motor control [1] and to generate a unified and accurate body representation [2]. For instance, distortions of body representation were reported when the processing of somatosensory information was altered with pain [3,4]. Individuals with chronic pain often report that painful body parts are larger [5,6,7], missing [5,8,9], or have the impression that these body parts do not belong to them and that they cannot move them [9,10]. Moreover, several studies show that, compared to pain-free controls, participants with chronic pain make movements that are slower [11,12] and less precise [13]. These motor deficits, accompanied by the alterations of body representation, suggest that sensorimotor integration may be altered in individuals with chronic pain.

To study sensorimotor integration alterations, a commonly used method is to expose subjects to experimental paradigms that induce a sensorimotor conflict since these conflicts disturb the use of afferent information [14,15,16,17,18,19]. In these experimental paradigms, participants perform a movement, while sensory information, such as visual feedback, contradicts the efferent and somatosensory information. In two recent studies performed in populations with chronic pain, participants pointed at targets while their arm was replaced by a virtual arm displayed in a 2D environment [20,21]. The virtual arm either followed the actual movement of the participants’ arm or displayed a reaching movement of smaller or larger amplitude (conflict conditions). Participants with fibromyalgia (FM; [21]) and with complex regional pain syndrome (CRPS; [20]) displayed a poorer ability to identify the direction of the conflict (smaller or larger amplitude) than the pain-free participants did. Motor performance during the conflict was either not assessed [20] or not altered in participants with chronic pain [21]. It should be noted, however, that the conflict used in these studies was subtle, and so perturbations of movement were relatively small. In studies using a more drastic sensorimotor conflict (e.g., visual feedback rotated by 180°), individuals with chronic pain reported more somatosensory disturbances and distortions of body representation, such as an increase in pain or the feeling of having an extra arm or losing an arm, in the conflict condition compared with the pain-free participants [17,22]. Despite these distortions, no alterations in motor performance [17] or motor adaptation [19] were observed during the conflict in participants with chronic pain. This dichotomy between perceptual deficits (i.e., reporting more somatosensory disturbances) and unaltered motor control in the presence of sensorimotor conflict could be hypothesized to result from a greater reliance on visual information, compared with somatosensory and efferent information, in individuals with chronic pain [18]. Overall, participants with chronic pain experience perceptual alterations in the presence of a sensorimotor conflict, as shown by body distortions and difficulties perceiving the conflict, but these alterations do not seem to be accompanied by motor impairments.

The study of the cortical response to sensorimotor conflicts in individuals with chronic pain could shed light on the discrepancy between perceptual deficits and the absence of motor impairments. However, to the best of our knowledge, the electrocortical activity of individuals with chronic pain has never been studied during a sensorimotor conflict; such studies have been performed exclusively on pain-free participants. They reveal that a sensorimotor conflict decreases the processing of afferent information, as expressed by reduced somatosensory-evoked potentials [23] and a decreased gamma power (≈30–100 Hz [15]) in the somatosensory cortex during the conflict. This has been suggested to reflect a sensory reweighting wherein somatosensory information is weighted less (and therefore less relied upon to guide movement) compared with visual information [15,24,25,26]. This could be a way for the brain to resolve the conflict by favoring one type of information (in this case, visual) over another. The attenuation (or suppression) of somatosensory information is accompanied by an improvement in motor performance (i.e., an adaptation to the conflict [15,27,28,29]), suggesting that reduced power in the sensorimotor cortex allows better motor performance during the conflict. In addition to the sensorimotor cortex, the posterior parietal cortex (PPC) also seems to play a role in adapting to conflict. Modulations of amplitude in gamma and theta power in the sensorimotor cortex and the PPC have been observed during the early stages (for gamma) and late stages (for theta) of adaptation to a sensorimotor conflict [30]. Furthermore, studies in pain-free individuals also suggest that the activity of the sensorimotor and posterior parietal areas may be modulated by conflict detection [31]. In a recent study, participants had to point at visual targets as quickly and accurately as possible. The visual feedback was delayed, creating a conflict that either was detectable (larger delays) or not detectable (smaller delays). The authors found an increased gamma power at electrodes pertaining to the sensorimotor cortex, which was greater when the conflict was detectable, and a decreased alpha power at posterior parietal electrodes, which was reduced when the conflict was detectable [31]. Overall, studies in pain-free individuals suggest that the oscillatory activity of brain areas, such as the sensorimotor and posterior parietal regions, during the sensorimotor conflict could be linked to conflict processing and motor performance. Assessing changes in the oscillatory activity of these brain areas (particularly in alpha, gamma, and theta frequency bands) is, therefore, of interest to understand the response to sensorimotor conflicts in individuals with chronic pain.

The general objective of this study was to assess the effect of sensorimotor conflict on motor performance and cortical activity in individuals with FM compared to pain-free controls. Fibromyalgia is a chronic widespread pain syndrome characterized by various symptoms, including fatigue, unrefreshing sleep, cognitive problems, and sensory alterations [32,33]. FM is also associated with a higher prevalence of several comorbidities, such as psychiatric disorders [34], irritable bowel syndrome, lupus, or chronic headaches [32,35]. Participants with FM and pain-free participants were exposed to a sensorimotor conflict, while cortical activity was recorded with electroencephalography (EEG). The first specific objective was to determine whether the behavioral performance differed between individuals with FM and pain-free controls. It was hypothesized that because of somatosensory alterations reported in individuals with FM, they would rely more on visual information and therefore show better performance when exposed to a sensorimotor conflict (i.e., a smaller difference between a condition with Congruent visual feedback and Incongruent visual feedback). The second specific objective was to explore the electrocortical response to the conflict in each group, which was performed by contrasting electrocortical activity between the Incongruent and the Congruent visual condition for each region of interest. Based on studies in pain-free subjects, we expected that the occurrence of sensorimotor conflict would result in changes in the gamma frequency band in the sensorimotor cortex and in the alpha and theta frequency bands in the PPC. Finally, the third specific objective was to compare the response to conflict (i.e., electrocortical response in the Incongruent condition only) between groups (FM vs. Control). No a priori hypothesis was made, given that no previous study explored this question.

## 2. Materials and Methods

### 2.1. Participants and Ethical Statement

A total of 20 adults with FM and 23 pain-free Controls matched for age and sex were recruited via the Fibromyalgia Association of Quebec City and Laval University for the individuals with FM and via Laval University and the FADOQ Network (a group of organizations for residents of Quebec who are 55 years old or older) for the controls. Sample size calculation was not performed because of a lack of quantitative reporting in EEG studies (mean, standard deviation, and effect sizes are often replaced by figures). The sample size was estimated according to previous studies showing statistically significant electrocortical differences during sensorimotor conflicts [15,23,36].

For all participants, inclusion criteria were as follows: (1) being 18 years old or older; (2) being right-handed (confirmed with the Edinburgh laterality inventory [37,38,39]; (3) having normal or corrected vision. Only right-handed participants were included because of differential activations in the left and right hemispheres during conflicts [40,41]. Participants with FM were included if (1) they had received a diagnosis of FM according to the American College of Rheumatology by a qualified doctor [32,42,43], (2) they had no motor impairments unrelated to FM that would interfere with the task performance (such as paresis or paralysis of the upper limb), and (3) they did not undergo surgery in the last three months. Exclusion criteria for control participants were the presence of a history of chronic pain (such as FM, chronic low back pain, or chronic headaches) and/or of acute pain severe enough to interfere with daily functioning in the last month or of acute pain on the day of the participation.

All participants provided their written informed consent prior to their participation in this study. The experiment was performed in accordance with the Declaration of Helsinki (except for registration in a database), and the study protocol was approved by the local ethical review board (Institut de réadaptation en déficience physique de Québec, Canada, no 2020-1858 RIS).

### 2.2. Study Design

Participants took part in one experimental session of about three hours at the *Centre de recherche interdisciplinaire en réadaptation et intégration sociale* (Cirris). All participants (FM and controls) first filled out questionnaires and underwent a tactile acuity assessment to identify potential somatosensory alterations in participants with FM. Additionally, individuals with FM completed clinical questionnaires. Then, all participants performed the sensorimotor task in which they were exposed to two conditions (Congruent and Incongruent). All trials started with an immobile phase to obtain a baseline electrocortical activity to normalize the activity during the other two conditions. Trials from the Congruent and Incongruent conditions were performed in random order.

### 2.3. Clinical Status and Tactile Acuity

Participants with FM were questioned about their medical history and asked to fill out the Brief Pain Inventory (BPI [44,45]) to assess the severity of pain and its impact on daily function. Tactile acuity was assessed in both groups with the two-point discrimination test (TPDT) [46]. A two-point esthesiometer was placed under its own weight (i.e., 10 g) on the participants’ skin of the ventral side of the index fingertip of the right hand, ensuring one or two simultaneous contact points while the participants’ eyes were closed. After each stimulation, participants were asked if they perceived one or two points. A series consisted of ten one-point stimulations and ten two-point stimulations, applied in random order. The initial distance between the two tips was 3 mm [47] and was decreased or increased gradually after each series, depending on the participant’s performance. The threshold was defined as the smallest distance between the two tips, which was perceived as two distinct stimuli at least 7 times out of 10. This measure was used to determine if participants with FM had alterations of somatosensory information, which could influence sensorimotor integration [27,48].

### 2.4. Instrumentation and Stimuli

**Figure 1:**
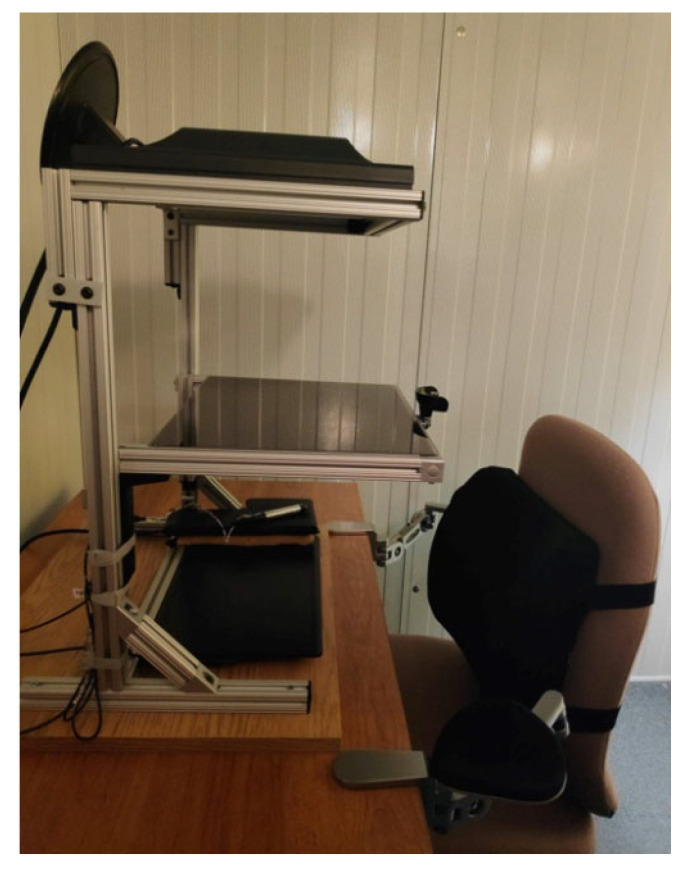
Experimental setup.

**Figure 2:**
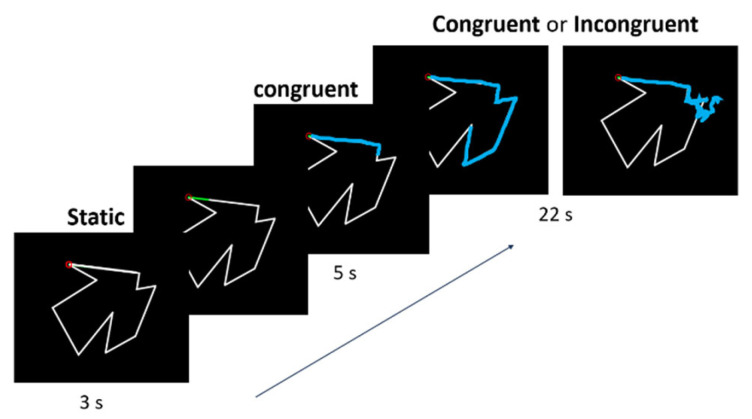
Time course of a single trial. The experiment (excluding familiarization trials) included a total of 48 trials, with 24 in the Congruent condition (veridical visual feedback) and 24 in the Incongruent condition (rotated visual feedback). In this last condition, half of the trials involved a 120° clockwise rotation and the other half a 120° counterclockwise rotation. The order of the trials in the two conditions and two directions of rotation was fully randomized.

The setup was composed of three levels (see Figure 1): an upper level with a computer screen (33.5 × 37.5 cm) facing downward, a middle level with a semi-reflecting glass, and a lower level with a digitizing tablet (20.3 × 32.5 cm; Wacom Intuos 4, Kazo, Japan) on the table. The glass was positioned at an equal distance between the screen and the digitizing tablet and reflected the virtual images of the screen that were used to complete the task on the tablet with a stylus. Since the set-up was in a dark room and the glass obstructed the vision of the participant’s hand, the only visual information available was the virtual images reflected by the glass. The participant’s task was to draw, as precisely as possible, the contour of sequentially presented two-dimensional shapes using the stylus held in their right hand. Visual feedback of the tip of the stylus was provided by a 3 mm grey square. The shapes were two irregular white polygons displayed on a black background. They consisted of 1–10 mm straight lines (10 angles) whose lengths varied between 31–90 mm (see Figure 2). The total perimeter was 186 mm for both shapes. The presentation of the visual stimuli and of the tracing trajectories was controlled by a custom MATLAB program (ver. R2020, MathWorks Inc., Natick, MA, USA) using the Psychtoolbox-3 [49].

### 2.5. Experimental Task

The time course of a trial is presented in Figure 2. Each trial started with the presentation of a shape and of the cursor. The participants had to place the cursor on the starting point—a red circle located at a randomly selected angle of the shape—and keep it still. After 3 s (period hereafter called the immobile phase), a 7 mm green segment indicated the direction to follow when tracing the contour of the shape. The participants were instructed to start tracing as soon as the segment appeared at a slow and constant speed (slow enough to draw about half of the shape). For 5 s, the visual feedback of the stylus (i.e., the cursor) remained veridical of the position of the tip of the stylus (congruent visual feedback). Then, for the remaining 22 s, the visual feedback either (1) continued to be veridical (Congruent condition, 24 trials) or (2) was rotated 120° clockwise or counterclockwise (12 trials for each rotation, randomized) compared to the actual stylus position (Incongruent condition). When the cursor left the shape’s contour, participants were instructed to bring it back to the point where it had left as quickly as possible and resume tracing. The 120° angle was chosen based on Lebar et al. [15], who determined that adaptation to sensory incongruence was the most difficult at this angle; it would therefore prevent a ceiling effect on motor performance. Moreover, the use of a design with rotations that were randomly applied clockwise or counterclockwise aimed to make the task more challenging and limit the speed of adaptation to obtain relatively stable conditions for the analysis of the cortical activity.

Frequent verbal feedback was provided between trials to ensure tracing speed was maintained slowly and constantly. This control of speed aimed to prevent large muscular artifacts and potential changes in neural oscillations [50]. It also allowed for reducing the speed of the ocular pursuit, which can contaminate EEG recordings. Two familiarization training were performed at the beginning of the experimental task (Congruent trials only) to ensure that the participants understood the instructions. The task consisted of 50 trials in total (2 familiarization trials and then 48 experimental trials with congruent or incongruent visual feedback) and lasted about an hour and a half, including breaks.

#### 2.5.1. Behavioral Measures

The stylus displacements were acquired at a sampling frequency of 30 Hz. The position of the stylus was low-pass filtered using a Butterworth filter (cut-off frequency = 4 Hz and order = 4). The (x, y) coordinates of the stylus position on the tablet were used to compute two complementary motor performance variables: (1) the radial error and (2) the number of zero-crossings [15,36]. The radial error was defined as the shortest perpendicular distance between the cursor position and the contour of the shape. The radial errors of each trial were averaged, and then the grand average across trials for each condition and for each participant was calculated. A large radial error means the position of the cursor was far from the contour of the shape, while a small radial error means the cursor was close to the contour of the shape. A sub-movement is composed of acceleration and deceleration phases. The transition from acceleration to deceleration is characterized by a zero-crossing, which is an instant at which the acceleration equals zero. Smooth tracing movements should contain a small number of zero-crossings [51]. Therefore, to quantify tracing smoothness, for each trial, the number of reversals in direction was calculated by computing the total number of zero-crossings in the resultant velocity time series of the cursor. This measure provides an estimate of the number of times the trajectory was corrected.

To control the effect of muscle contraction on electrocortical activity, the grip force applied to the stylus during the task was measured in mV with a strain gauge located where the fingers naturally sit on the stylus. To assess whether the force applied in the stylus was different in each condition and group, changes in strength of 2000 ms after conflict onset compared with 2000 ms before conflict onset were expressed with the Root Mean Square (RMS) value, from which the baseline voltage (i.e., voltage when the stylus is on the table, with no contact with the fingers) was subtracted.

#### 2.5.2. Electrocortical Activity Measures

Electrocortical activity was recorded with a 64-electrode HydroCel™ Geodesic Sensor Net (Electrical Geodesics Inc., Eugene, OR, USA) and amplified with the Net Amps 400 amplifier (Electrical Geodesic Inc., Eugene, OR, USA). EEG recording was sampled at 1000 Hz using Net station 5.4 software (EGI, Eugene, OR, USA), and electrode impedances were kept below 50 kΩ. The size of the sensor net was chosen to match the participants’ head size. A microcontroller (Arduino Uno, Atmel, Corporation, San Jose, CA, USA) synchronized EEG data with specific temporal events (i.e., trial onset and offset and change from Congruent to Incongruent condition).

EEG data were processed offline using custom MATLAB scripts (ver. 2019b) and EEGLAB 2021.1 [52]. For the preprocessing, EEG signals were down-sampled to 250 Hz, and then a band-pass filter was applied between 1 Hz and 100 Hz, and power line noise and its harmonics were removed using the EEGLAB plug-in CleanLine and ZapLine. CleanLine (which uses a non-stationary temporal filter) and ZapLine (which uses a stationary spatial filter). These two algorithms improve line-noise cleaning since they are complementary [53]. Clean_rawdata, another EEGLAB plug-in, was subsequently used to detect and subtract artifacts originating from eye blinks, muscles movement, or electrode motion, and to interpolate or reject segments of the signal that exceeded (or electrodes whose signal exceeded) the mean amplitude by four standard deviations of a clean portion of the same data. The electrode signals were then re-referenced against the average of the activities recorded by all electrodes. For both experimental conditions, trials lasted 30 s in total. For the Incongruent condition, the conflict occurred 8 s after trial onset (i.e., after 3 s of immobility and 5 s of congruent visual feedback). After preprocessing, the EEG data epochs were realigned such that the onset of the conflict was set to time = 0 s. EEG data were segmented into epochs from −4 to +4 s with respect to conflict onset. For the Congruent condition, EEG epochs were also realigned with a temporal marker at 8 s following trial onset. The period between −2 and −0.2 s served as a baseline, and the period between 0 and 3 s was used for conditions comparison. Epochs containing large artifacts were rejected through visual inspection.

**Figure 3:**
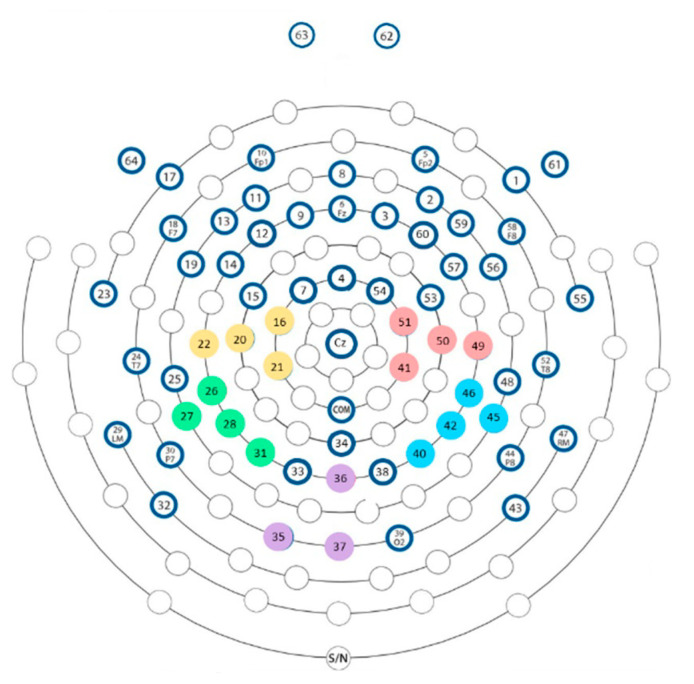
Electrodes are chosen as part of each region of interest. The cluster of electrodes over the left sensorimotor cortex is in yellow, the cluster for the right sensorimotor cortex is in pink, the cluster for the left PPC is in green, the cluster for the right PPC is in blue, and the cluster for the visual cortex is in purple.

For each participant and each cortical region of interest (i.e., visual, left and right PPC, and left and right sensorimotor areas), the electrode with the larger difference of alpha power in the Incongruent condition compared with the Congruent condition was selected. The electrodes chosen for each region of interest are shown in Figure 3. For each selected electrode, the time–frequency map was calculated by multiplying the power spectrum of the electrode calculated from the fast Fourier transform by the power spectrum of complex Morlet wavelets [54]. The Morlet wavelets were defined as $e^{i2\pi tf}e^{-t^{2}/\left(2\sigma^{2}\right)}$, where t is the time; f is the frequency, which increased from 1 to 50 Hz in 60 linearly spaced steps; and σ is the width of each frequency band. The Morlet wavelets were set according to $n/\left(2\pi f\right)$, where n is the number of wavelet cycles, which increased from 4 to 12 in linearly spaced steps. Then, the inverse fast Fourier transform (i.e., frequency domain convolution) was performed. From the resulting complex signal, an estimate of frequency band specific power at each time point was defined as the squared magnitude of the result of the convolution $Z\left(real{\left[z\left(t\right)\right]}^{2\;}+imag\left[z\left(t\right)\right]{\;}^{2}\right)$. Power was normalized using a decibel (dB) transform ($dB\;power=10\times log10\;\left[power/baseline\right]$), where the baseline was the average power at each frequency band from −2 s to −0.2 s before time 0, averaged across conditions. Conversion to dB ensures that data across all frequencies, time points, electrodes, conditions, and participants are on the same scale and thus comparable. The frequency bands analyzed were theta, known in particular for its role in the detection of errors [55,56,57] and adaptation to conflict [30]; alpha, whose increase has been associated with rest and cortical deactivation [55,58]; beta, implicated in motor control [55,56,59]; and gamma, linked to sensorimotor integration [55,58].

### 2.6. Statistics

#### 2.6.1. Clinical and Demographic Data

Since the distribution of the TPDT threshold was not normal, the comparison was performed with Mann–Whitney–Wilcoxon’s U, using IBM SPSS Statistics (ver. 29), with a significance threshold at *p* < 0.05. Other clinical and demographic data were synthesized with descriptive statistics. Note that because nonparametric statistics were used, the descriptive statistics reported include median and interquartile range (IQR).

#### 2.6.2. Behavioral Data

Since none of the data followed a normal distribution (as shown by a significant Shapiro–Wilk test), and transformations did not resolve the skewness of the data, nparLD, a non-parametric equivalent of a repeated measures ANOVA [60], was performed on all kinematic variables, with the within-subject factor Condition (Congruent and Incongruent) and the between-subject factor group (FM and Control) (specific objective #1). NparLD is a robust method for mixed designs with inequivalent samples and does not require normality of distributions and homoscedasticity [60]. NparLD was performed with RStudio Team (2023). Note that because nonparametric statistics were employed, the descriptive statistics reported include median and IQR. The effect sizes were expressed as the difference between the relative treatment effects for each modality that was compared. The bigger the difference between relative treatment effects, the larger the effect size is (see [61]; a difference around 0.11 is considered small, and a difference around 0.43 is considered large). Outliers (as defined as values outside of 2.5 times the interquartile range) were excluded from the data.

For the Incongruent condition, a comparison of the motor performance in the first six trials and the last six trials of each group was performed to verify whether sensorimotor adaptation to the conflict was similar between groups (methodological control for analyses on electrocortical activity measures). Results were considered significant for *p* < 0.05.

#### 2.6.3. Electrocortical Activity Measures

T-tests were performed on the time–frequency data, and multiple comparisons were corrected with cluster-based permutation testing [62,63]. First, a distribution of maximal cluster sizes under the null hypothesis was obtained with permutation testing, which consists of randomly shuffling the attribution of condition for each data point and recomputing statistics each time. After thresholding each permutation map (*p* < 0.05), the t-values were stored. This was repeated 1000 times to create a distribution of t-values under the null hypothesis. Then, the real data was compared to the data under the null hypothesis to see if there were any significant differences: any cluster in the real data with a t-value larger than 97.5% of the distribution of the null hypothesis was considered statistically significant [62,63]. The permutation test makes it possible to test the null hypothesis (H0), which states that the data in the experimental conditions (Incongruent vs. Congruent for specific objective #2; FM vs. Control for specific objective #3) come from the same probability distribution. Thus, the following significant result means that H0 can be rejected: the alternative hypothesis (H1) is supported, suggesting that the data come from different distributions and is, therefore, different between conditions. Overall, cluster-based permutation tests indicate whether there is a significant difference between conditions. Since permutation tests result in a normal data distribution (even though they are technically non-parametric tests), the reported descriptive statistics for the electrocortical measures include mean and standard deviation.

First, this method was performed to test the differences between conditions (Incongruent vs. Congruent) in each group separately for each chosen electrode to confirm that the Incongruent condition was associated with differences in electrocortical activity (specific objective #2). The complete time window was used for this analysis.

Second, the same statistical method was used to explore the differences between groups (FM vs. Control) in the Incongruent condition only (specific objective #3), but this time over specific time periods that were relevant to each frequency band based on the first analysis (contrast between the Congruent and Incongruent conditions) for theta frequency band (4–8 Hz), [0; 800] ms; for alpha frequency band (8–12 Hz), [500; 3000] ms; for beta frequency band (15–30 Hz), [500; 3000] ms; and for gamma frequency band (35–60 Hz), [500; 3000] ms. An increase in power in each frequency band means there is synchronization of the underlying neuronal population in this frequency band, whereas a decrease in power reflects a desynchronization [58]. Preprocessing of the data removed an average of 7.0% of the total trials for the FM group and an average of 12.2% for the Control group. NparLD showed no significant difference in the number of trials removed in each Group (F(1, 25) = 0.93, *p* = 0.37) and each Condition (F(1, 25) = 1.32, *p* = 0.25), and no interaction effect (F(1, 25) = 0.01, *p* = 0.90).

## 3. Results

In total, the data of 43 participants (20 participants with FM and 23 controls) were included in the behavioral analysis, and two trials were removed because of technical issues. A subsample of 25 participants (12 FM and 13 controls) performed the task as their electrocortical activity was recorded.

The FM group and the Control group were both composed exclusively of female participants who were of similar age (FM: median = 46.5, IQR = 19.5 years old; Control: median = 44, IQR = 25.5 years old). Mann–Whitney–Wilcoxon’s U showed no statistical difference in the TPDT threshold between the two groups (3 ± 2 mm for the Control group and 3 ± 1 mm for the FM group, *p* = 0.39). The clinical profile of the FM group is described in Table 1. For this group, the BPI scores indicated a median pain severity of 4.8/10 (IQR = 0.7) and a median pain interference with daily living of 4.9/10 (IQR =1.3). 

### 3.1. Behavioural Results

#### 3.1.1. Results of the Complete Sample

**Figure 4:**
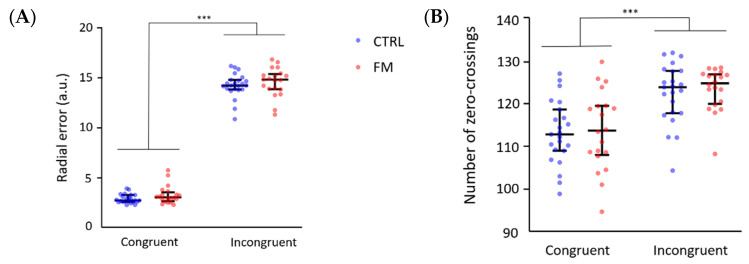
Differences in (**A**) the radial error and (**B**) the number of zero-crossings. Each point represents the mean for each participant, and horizontal and vertical bars represent the median and IQR (respectively) for each group. CTRL = Control participants; *** indicates a *p* < 0.001; other differences are not significant.

Results are presented in Figure 4. Sixteen trials were excluded (in a total of 48 trials×43 participants=2064 trials) because they were outliers with respect to the radial error or the number of zero-crossings. For the grip force, the data of one participant (S32) were excluded because of technical issues; hence, the analysis was performed on 42 participants.

We first compared the tracing speed and the grip force applied on the stylus between the groups to determine whether the task was performed similarly (results not shown; methodological controls). The nparLD test showed that participants were faster under Incongruent (median = 28.18, IQR = 15.87 a.u.) compared with Congruent visual feedback (median = 24.09, IQR = 4.67 a.u.; F(1, 42) = 42.92, *p* < 0.001, effect size = 0.27), but there was no significant difference in speed between Control participants and participants with FM (F(1, 42) = 1.20, *p* = 0.27), and there was no Group–Condition interaction (F(1, 42) = 0.00, *p* = 0.98). For changes in grip force during the conflict, the nparLD test revealed no effect of Group (F(1, 41) = 0.04, *p* = 0.85) or Condition (F(1, 41) = 0.28, *p* = 0.59), and no interaction (F(1, 41) = 0.28, *p* = 0.59).

The nparLD tests performed to test the specific objective #1 showed a higher radial error in the Incongruent condition compared with the Congruent condition (F(1, 42) = 321.82, *p* < 0.001, effect size = 0.51), but no effect of the Group (F(1, 42) = 2.11, *p* = 0.15) and no interaction (F(1, 42) = 0.05, *p* = 0.81) (see Figure 4A). The same pattern of results was observed for the number of zero-crossings, with a significant effect of the Condition (F(1, 42) = 51.77, *p* < 0.001, effect size = 0.32) and no effect of the Group (Group: F(1, 42) = 0.43, *p* = 0.51) or interaction (F(1, 42) = 0.02, *p* = 0.88) (see Figure 4B). Unsurprisingly, these results confirm a poorer motor performance when exposed to a conflict between visual and proprioceptive information. However, the performance did not differ significantly between participants with FM and controls for both conditions. No adaptation to the conflict was found for either group. Indeed, no effect of the type of trials (first six trials, last six trials) was found for the radial error (F(1, 42) = 1.95, *p* = 0.16) or the number of zero-crossings (F(1, 42) = 2.50, *p* = 0.11) and no interaction type of trial X Group was observed for the radial error (F(1, 42) = 1.83, *p* = 0.18) or the number of zero-crossings (F(1, 42) = 0,47, *p* = 0.49).

Overall, these results show no significant difference in the mean performance between groups, whether in Congruent or in Incongruent conditions. Moreover, the lack of adaptation to the conflict is likely due to the randomized presentation of trials without conflict and trials with either clockwise or counterclockwise visual rotations. Importantly, this lack of adaptation to the sensory conflict allowed us to pool all trials for the analysis of electrocortical activity measures (specific objectives #2 and 3; see Section 3.2).

#### 3.1.2. Results of the Subsample of Participants for Which Electrocortical Activity Was Recorded

We present the behavioral results for the subsample of participants (12 FM and 13 controls) who performed the task while having their electrocortical activity recorded. The results (not shown) are similar to the full sample but with less statistical power due to the smaller sample size. The nparLD revealed that participants were faster under Incongruent compared to Congruent visual feedback (F(1, 24) = 35.39, *p* < 0.001, effect size = 0.26), but there was no difference in speed between Control participants and participants with FM (F(1, 24) = 1.34, *p* = 0.25) and no Group–Condition interaction (F(1, 24) = 0.000009, *p* = 1.0). For the changes of grip force at conflict onset, the nparLD test revealed no effect of Group (F(1, 24) = 0.0007, *p* = 0.98) or Condition (F(1, 24) = 1.36, *p* = 0.24), and no interaction effect (F(1, 24) = 1.83, *p* = 0.18).

The nparLD tests showed a higher radial error in the Incongruent condition as compared with the Congruent condition (F(1, 24) = 260.67, *p* < 0.001, effect size = 0.50), but no effect of the Group (F(1, 24) = 0.37, *p* = 0.55) and no interaction (F(1, 24) = 3.31, *p* = 0.07), although there was a trend towards a greater performance deterioration in the Incongruent condition for the Controls. For the number of zero-crossings, we found a significant effect of the Condition (F(1, 24) = 32.67, *p* < 0.001, effect size = 0.31) and no effect of the Group (Group: F(1, 24) = 1.06, *p* = 0.30) or interaction (F(1, 24) = 0.76, *p* = 0.38). Unsurprisingly, these results confirm a poorer motor performance when exposed to a conflict between visual and proprioceptive information. Similar to the results of the full sample, the performance was not statistically different between participants with FM and controls for both conditions.

### 3.2. Electrocortical Activity Results

**Figure 5:**
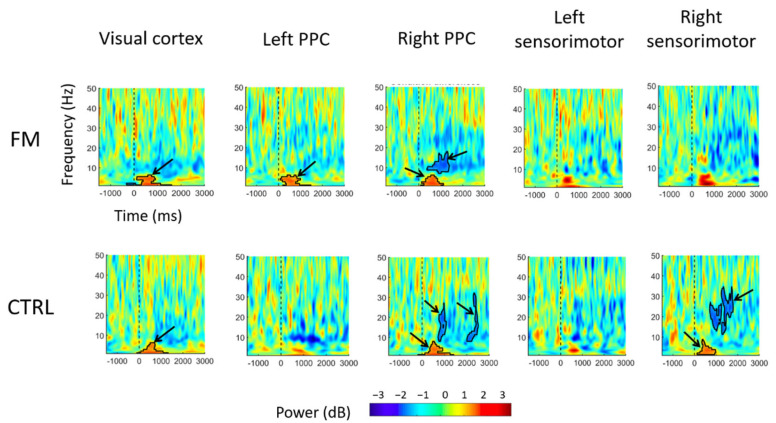
Time–frequency map differences for the Incongruent condition compared with the Congruent condition. The two rows represent differences in activation in the Incongruent condition compared with the Congruent condition for each group (FM and CTRL, respectively). A positive power (represented by warm colors, as indicated in the power bar) means an increase in power (i.e., synchronization), while a negative power (cold colors) reflects a decrease in power (i.e., desynchronization). The frequency bands can be defined as follows: theta (≈4–7 Hz), alpha (≈8–12 Hz), beta (≈15–30 Hz), and gamma (≈35–60 Hz). Bold lines enclose regions of continuous pixels that were significantly different from the Congruent condition (see black arrows). CTRL = Control participants.

The time–frequency maps of the contrast between the Congruent and the Incongruent conditions for each group and each region of interest (visual cortex, left/right PPC, and left/right sensorimotor cortex) are shown in Figure 5.

When looking at each group independently across the complete time window (0 to 3000 ms), the cluster-based permutation test indicated that significant differences across conditions could be observed as follows (outlined by bold lines in Figure 5):

- *Visual cortex*: For both groups, a significant increase in theta (4–7 Hz) power in the Incongruent condition was observed within the first 1000 ms following sensorimotor conflict onset, compared with what was observed in the Congruent condition.
- *PPC*: In the left PPC, an increase in theta power was observed only for the FM group between 0 and 1000 ms after the onset of sensorimotor conflict in the Incongruent condition compared with the Congruent condition, whereas no significant difference was found for the Control group.
  In the right PPC, theta power increased between 0 and 1000 ms after conflict onset for both groups. A decrease in alpha power (8–12 Hz) was observed, from approximately 300 to 1200 ms after conflict onset for the FM group and from between 300 and 800 ms after conflict onset for the Control group. A decrease in beta power was also observed, with a cluster extending from approximately 2000 to 2500 ms after conflict onset in the Control group.
- *Sensorimotor cortex*: In the right sensorimotor cortex (ipsilateral to the tracing hand), there was a significant increase in theta power (a cluster extended from ~200 to ~1000 ms) after conflict onset, followed by a decrease in beta power (a cluster extended from ~500 to ~2000 ms), only for the Control group.

**Figure 6:**
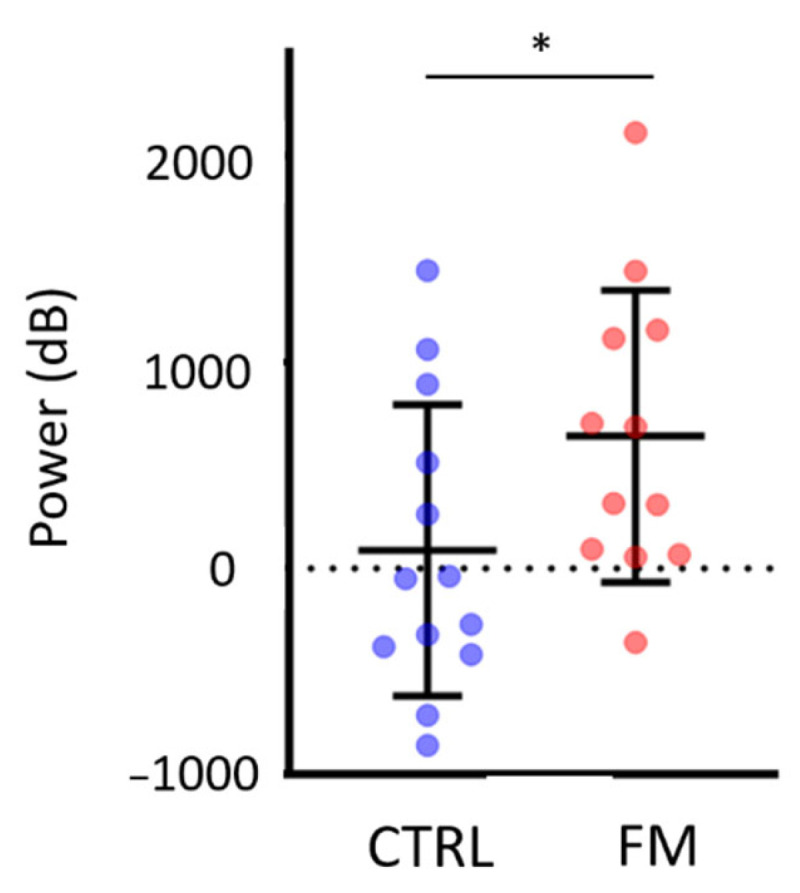
Group difference in theta power over the left PPC in the Incongruent condition (time window = [0–800] ms). Each dot represents the mean for a participant, and horizontal and vertical bars represent the mean and the standard deviation (respectively) for each group. The asterisk (*) indicates a significant group difference (i.e., *p* < 0.05).

When comparing the electrocortical activity across the groups in the Incongruent condition for specific time periods that were relevant for a given frequency band (theta: 0 to 800 ms; alpha, beta, and gamma: 500 to 3000 ms), the only statistically significant difference was found in the left PPC: the amplitude of theta power after conflict onset was higher in the FM group compared with the Control group (*p* = 0.029). This is shown in Figure 6.

## 4. Discussion

At the behavioral level, results show a degradation in motor performance in the Incongruent condition compared with the Congruent condition; this is similar across both groups. No substantial adaptation to the conflict over time was observed. The analysis for changes in electrocortical activity between the Incongruent and Congruent conditions (Figure 5) revealed that conflict onset was marked by a general increase in theta power in the visual cortex, which confirms that both groups visually detected the conflict. Comparisons between the Incongruent and Congruent conditions showed significantly larger power in the theta band over the left and right parietal cortex for the FM group and only over the right parietal cortex for the Control group. Group comparison confirmed that the conflict caused a larger increase in theta band power over the left parietal cortex for the FM group (Figure 6). With the Congruent condition, shortly after conflict onset, a decrease in alpha power in the right PPC was observed in both groups, followed by a decrease in beta power in the right PPC for the Control participants. A decrease in beta power was also observed over the right sensorimotor cortex in Control participants. The absence of group differences for the behavioral variables will first be examined; then, EEG results will be discussed. The dissociation between altered perception and unaltered motor control during exposure to sensorimotor conflict in individuals with FM will then be interpreted. Lastly, some limitations of this study will be outlined.

### 4.1. Effect of the Sensorimotor Conflict on the Behavioral Measures

Our first specific objective was to determine whether the behavioral performance differed between individuals with FM to the performance of pain-free Controls. As expected, the sensorimotor conflict disturbed movement, and participants made more motor errors with biased visual feedback. Sensory and motor information is generally congruent in daily life. Consequently, being exposed to an incongruence between visual information and somatosensory/motor information will temporarily lead to more errors until the nervous system changes its strategy and adapts [15,23,36,48,64,65]. Contrary to findings in several previous studies [15,23,36,48,66], no motor adaptation during the conflict was found in either group. Our behavioral paradigm was specifically designed to be challenging and limited the speed of adaptation (e.g., it unpredictably varied between clockwise and counterclockwise visual rotation [67], and the amplitude of the rotation was high (±120°) [67]). The stability of motor performance allowed us to pool the results of all trials for the analysis of cortical responses. However, we were still expecting some adaptation, considering Lebar et al. (2017) [15] did show an adaptation to the conflict used in this present study in healthy volunteers. The fact that we did not observe significant improvement over time in the Incongruent condition might be explained by the fact that our participants were older (mean age of 46 years old in our study vs. 26 years old in Lebar’s study [15]) and/or that there were fewer trials (half the number used in Lebar’s study), due to the fatigability of the FM population. The high level of difficulty of the task, which was optimized for the EEG analyses, might also contribute to explaining why, contrary to our initial hypothesis, no differences were observed between groups at the behavioral level. Future research should focus on behavioral measures if only to avoid this constraint since Bultitude et al. (2016) [68] hypothesized that a large sensorimotor conflict could reduce or prevent the integration of sensory and motor information. According to this view, when the discrepancy between visual and somatosensory/motor information is too large, the central nervous system infers separate origins for the signals (i.e., the drawing errors seen through the visual feedback are not generated by the motor system) and so processes them separately, without correcting for the motor errors.

### 4.2. Effect of the Sensorimotor Conflict on the Electrocortical Activity

Specific objective #3 was to compare the response to conflict (i.e., electrocortical response in the Incongruent condition only) between groups (FM vs. Control). No a priori hypothesis was made given that no previous study explored this question. The only difference between groups was found in theta power, and therefore, theta power will be discussed first (for both specific objectives #2 and #3). Results show an increase in theta power at the onset of conflict, over the sensorimotor, posterior parietal, and occipital cortices in both FM and Control groups. Similar modulation of theta has frequently been observed during sensory conflicts, especially in regions pertaining to the frontoparietal network [30,69,70,71,72,73,74,75]. In a sensorimotor conflict, a modulation of theta power slightly anticipated movement correction and was influenced by the amplitude of the motor error [75]. This modulation is commonly interpreted as a general error processing mechanism, encompassing error detection and error correction [70,71,73,76,77,78,79,80]. According to the internal model theory, the detection of error arises when a discordance occurs between the actual state of the sensorimotor system, estimated by the sensory afferences, and its predicted state [81,82]. This implicit process is essential for motor adaptation [64,83,84]. In contrast, the correction of error originates from comparing the desired state of the system and its estimated actual state [81,82]. The correction of error also plays a key role in the strategic modification of the motor command to minimize error [85,86,87,88]. These processes could partly rely on theta oscillations. In an attempt to disentangle error detection from error correction, Savoie and colleagues (2018) compared the electrocortical activity of participants who were explicitly taught to counter a sensorimotor conflict with that of participants who had already adapted to the conflict. The authors found an increase in theta power at posterior parietal electrodes in the first group of participants, which suggests a particular role of parietal theta oscillations in detecting conflict for subsequent adaptation [30]. This result is corroborated by findings that show clear deficits of motor adaptation in patients with posterior parietal lesions, with no impairment in online error correction [89,90]. In this present study, the increase in theta power was observed over the sensorimotor, posterior parietal, and visual cortices, suggesting the detection of the incongruence between visual, somatosensory, and motor information. These regions are typically involved in the processing of visual information (visual cortex; [91]), somatosensory information (somatosensory cortex; [92]), and their subsequent integration (PPC; [93]).

Importantly, the increase in theta power over the left PPC was only present in participants with FM, in contrast to Control participants. This difference could indicate a stronger discordance between the predicted state of the sensorimotor system and the actual state estimated by the sensory afferences in individuals with FM. Considering that the motor performance of FM and Control participants was disturbed by the conflict, it is unlikely that the sensory consequences estimated by afferences were more altered in the FM group than in Controls [21] (notably, no alteration in TPDT threshold was found in our sample, consistent with the results of a recent meta-analysis [94]). Therefore, the predicted state generated by the predictors (also referred to as the forward model) might be impaired in the FM participants. This result is in accordance with a recent study showing sensory disturbances during a sensorimotor conflict in participants with FM and controls, which were stronger in FM participants during active movement only but not during passive movement (i.e., no predicted state involved). Moreover, no difference in motor perturbations was found between the groups [17].

Besides changes in theta power, results related to specific objective #2 showed a response to sensorimotor conflict, without group differences, in alpha and beta power. More specifically, a decrease in alpha and beta power was observed over the PPC, and a decrease in beta power was observed over the sensorimotor cortex. Overall, the results are consistent with our hypothesis in terms of regions involved in the response to conflict (sensorimotor cortex and PPC) but not completely in terms of frequency bands involved. This can be explained by the fact that the tasks that have been used in the few studies on electrocortical activity during sensorimotor conflicts are quite different from each other (and so the hypotheses put forward would be based on limited evidence). The observation of a decrease in alpha over right PPC, starting at 300 ms after conflict onset for both groups, further supports the involvement of the PPC in conflict detection. This decrease, which occurs rather simultaneously with the theta power increase, could reflect an increase in the local excitability of the neuronal population with respect to incoming afferent information [56]. This alpha modulation is thought to reflect an attentional (top-down) modulation of cortical excitability related to the enhancement of task-relevant information (i.e., visual here) or suppression of irrelevant information (i.e., proprioceptive here) [95,96]. Finally, the decrease in beta power found over the sensorimotor cortex could be related to higher processing of somatosensory information in the Incongruent condition [15,97,98]. A possible explanation for this modulation is the higher tracing speed in this condition. It has been shown that the use of somatosensory information is favored over visual information during rapid movements because of its superior transmission speed [1]. In our study, frequent feedback was given about speed to control for this variable, but participants of both groups still drew faster in this condition compared with the no-conflict condition [15]. This increase in speed could reflect the participants’ wish to quickly bring the cursor back to the point it left the polygon and rapidly correct their trajectory.

Contrary to our hypothesis, we found no difference in gamma-band power in the Incongruent condition compared with the Congruent condition. This contrasts with studies that show a decrease in gamma power during sensorimotor conflicts [15,25]. The lack of change in our study could be linked to the absence of adaptation in the Incongruent condition. Lebar et al. (2017) hypothesized the role of decreased gamma power in reducing the weight of somatosensory information, thus improving performance during a sensorimotor conflict [15]. The fact that we did not observe any motor improvement during the conflict in our study could therefore suggest a steady contribution of somatosensory inputs throughout the conflict trials. Another explanation could be the poorer signal-to-noise ratio in higher frequencies because power decreases with frequency [58]. This makes significant differences in gamma power harder to observe.

### 4.3. Clinical Implications

This present study focused on the impact of chronic pain (FM) on the effect of sensorimotor conflict on motor performance and electrocortical activity. Because individuals with FM or other types of chronic pain have been shown to report higher somatosensory perturbations in response to conflicts [14,22] and have also been shown to have an impaired ability to detect feedback manipulation [20,21], we expected to see differences between groups, especially in brain activity related to conflict perception. The results are rather consistent with observations from recent studies that show that motor performance in chronic pain individuals is similar to or even better than [19] that of pain-free individuals [18,19,21]. Our results can be interpreted in a theoretical framework that proposes two distinct visual pathways: one mediating conscious perception (ventral stream) and the other guiding motor action (dorsal stream) [99]. One of the lines of evidence for such dissociation comes from research on the effect of visual illusions on reaching and grasping behavior. Although the two visual systems theory has been challenged and alternative mechanistic explanations have been proposed, there is still ample evidence that at the behavioral level, perception is prone to visual illusion while action remains immune to it [100]. However, these dissociations may depend critically upon the stimuli used and the response conditions used [101]. While the paradigms in those dissociation studies are very different from the ones that have been used in studies of individuals with chronic pain, these observations may help us understand the apparent discrepancy between the presence of substantial perceptual alterations and the absence of motor alterations during exposure to sensorimotor conflict in individuals with chronic pain [14,19,21,22].

### 4.4. Limitations

This study has some limitations. First, the participants were only women, so the results are not generalizable to men. This is because, even though the more recent diagnostic criterion for FM affects women and men in similar proportions [102] and sex was not an exclusion criterion in this study, the sample reflects the overall higher proportion of women in this population, most of whom were diagnosed with older criteria. Second, we did not test whether proprioception was altered in the FM group. The hypothesis would be that if proprioception is altered in participants with FM, it would be weighted less than visual information during the sensorimotor conflict [18,103]. Therefore, participants with FM would be less affected by the conflict [103], which might result in an attenuated cerebral response to conflict and fewer motor errors during the conflict compared with pain-free participants. Since studies assessing proprioception in individuals with FM report conflicting results [104,105,106,107], we can only assume that, in light of our results (i.e., similar motor performance between FM and Control groups), it is likely that no difference in proprioception would have been found between our groups. Third, electrocortical activity was recorded with EEG for a subsample of participants (25 out of 43). Some participants with FM had trouble tolerating the EEG net, so we chose to only measure behavior in these participants. It should be noted that the EEG net is both wet and relatively tight, two factors that can trigger or accentuate pain in FM [108,109]. Since we found similar behavioral results in the subsample and the whole sample of participants, it is unlikely that the subsampling introduced any bias into our data. (Moreover, several EEG studies on sensorimotor adaptation have similar sample sizes per group [23,36,66].) Finally, the electrocortical activity was analyzed at the electrode level and not at the source level, which provides less precise spatial information and could explain the presence of brain modulations in the ipsilateral hemisphere. That said, modulations of the activation of parieto-occipital and sensorimotor cortices have been observed in previous studies involving movement and integration of somatosensory information and could reflect transcallosal inputs from the contralateral hemisphere [58,110,111,112]. Moreover, the EEG data is still in accordance with previous research on conflict detection, which validates our methodology.

## 5. Conclusions

In conclusion, the presence of conflict was associated with worse performance for both FM and Control participants (a significant increase of 400% for the radial error and 10% for the number of zero-crossings, compared with the condition with no conflict). Results suggest the presence of a stronger error detection signal, as shown by a significant increase in power in the theta frequency band over the left posterior parietal cortex in the FM group. Thus, despite this stronger signal and the somatosensory perturbations observed in individuals with FM during the sensorimotor conflict, motor performance does not seem to be more altered in this population, compared with pain-free individuals. This points to a dissociation between an altered perception of action and a seemingly unaltered control of action in individuals with fibromyalgia.

## Figures and Tables

**Table 1:** Clinical profiles of participants with FM.

Participant	Sex	Age (years)	Currently Working?	Pain Duration (years)	BPI: Pain Severity	BPI: Pain Interference	Pharmacological Treatments	Non-Pharmacological Treatments	Current Comorbidities
S02	F	66	no	32	4.8 ± 0.8	6.4 ± 1.2	Acetaminophen, tridural, tramacet, amitriptyline, gabapentine		Migraines
S04	F	56	no	11	6.5 ± 1.7	3.4 ± 3	Naproxen	Physiotherapy, osteopathy	Hypothyroidism, coeliac disease
S06	F	32	yes	7	3.5 ± 1.7	1.6 ± 1.2	Amitriptyline, duloxetine, bisoprolol	Psychotherapy, massage, acupuncture, TENS	Tachycardia, chronic fatigue syndrome
S14	F	59	no	3	7 ± 0.7	5.6 ± 2.8	Venlafaxine	Meditation	Epstein-Barr virus, irritable bowel syndrome
S15	F	20	yes	3	4.8 ± 1.1	5 ± 1.7	Diclofenac, duloxetine	Psychotherapy, acupuncture, massage, nutritionist	Restless leg syndrome, arthritis, irritable bowel syndrome, migraines, generalized anxiety disorder, borderline personality disorder, eating disorder, attention and hyperactivity disorder, triple X syndrome
S16	F	34	yes	21	4 ± 1.2	2.1 ± 2.6	Cyclobenzaprine	Psychotherapy	Arthritis, borderline personality disorder, migraines, endometriosis, post-traumatic stress disorder, hyperlaxity
S21	F	64	no	12	4.8 ± 0.8	4.6 ± 1.8	Acetaminophen, codeine	Physiotherapy	Eczema, asthma, irritable bowel syndrome, arthritis, Raynaud’s disease
S39	F	52	no	10	3 ± 1.2	0.6 ± 0.9	Pregabalin, acetaminophen	Massage, chiropractic	Aerophagia
S01	F	45	yes	31	5.5 ± 1.5	5.4 ± 1.8	Pregabalin, naproxen, amitriptyline, escitaloprame	Physiotherapy, meditation	Slipped disc, hypothyroidism, chronic fatigue syndrome, kinesiophobia
S03	F	51	yes	46	4.5 ± 0.9	5 ± 1.9	None	Physiotherapy, psychotherapy	Biliary cirrhosis, hypothyroidism, generalized anxiety disorder, post-traumatic stress disorder, type 2 diabetes, obesity, sleep apnea, asthma, migraines, chronic fatigue syndrome, depression
S05	F	21	yes	9	4 ± 0.7	0.9 ± 0.8	Aventyl	Physiotherapy, massage	Irritable bowel syndrome, migraines
S08	F	23	yes	5	5.3 ± 2.3	3.6 ± 2.8	Acetaminophen, cyclobenzaprine	Massage	Attention disorder, post-traumatic stress disorder
S11	F	39	no	39	1.5 ± 1.1	1.6 ± 1.2	None	Chiropractic, osteopathy, massage	
S12	F	51	no	6	5.8 ± 1.8	7 ± 1.2	Flexeril	Massage	Depression
S13	F	41	yes	41	7 ± 0.7	5.1 ± 3.1	Pregabalin, celebrex, flexeril, cannabis	Psychotherapy, physiotherapy, osteopathy	Depression, rhumatoid arthritis, irritable bowel syndrome, migraines
S17	F	48	no	10	5 ± 2	5.3 ± 1.7	Restoril	Psychotherapy, osteopathy, physiotherapy, acupuncture	Sclero-atrophic lichen
S19	F	48	no	9	4.3 ± 1.5	5.4 ± 2.6	None	Osteopathy	
S22	F	24	yes	10	4.8 ± 1.1	4 ± 2.4	Pregabalin	Osteopathy	Hyperactivity disorder, hypothyroidism, irritable bowel syndrome, asthma, migraines
S23	F	37	yes	12	6 ± 1	4.9 ± 1.4	Ibuprofen, acetaminophen, decontractyl	Massage, chiropractic, osteopathy	Type I diabetes
S26	F	66	no	21	4 ± 1.6	4.9 ± 2.4	None	Psychotherapy, physiotherapy, meditation	Irritable bowel syndrome, osteoporosis, chronic rhinitis
**Median ± IQR**				**10.5 ± 15**	**4.8 ± 1.6**	**4.9 ± 2.3**			

The grey box includes the participants with FM for whom EEG data during the sensorimotor task is available.

## Data Availability

The data presented in this study are available upon request from the corresponding author. The data are not publicly available due to restrictions related to ethical approval.
